# Funding Sources of Therapeutic and Vaccine Clinical Trials for COVID-19 vs Non–COVID-19 Indications, 2020-2021

**DOI:** 10.1001/jamanetworkopen.2022.26892

**Published:** 2022-08-16

**Authors:** Aris Angelis, Carlos Suarez Alonso, Ilias Kyriopoulos, Elias Mossialos

**Affiliations:** 1 Department of Health Services Research and Policy, London School of Hygiene and Tropical Medicine, London, United Kingdom; 2 Department of Health Policy and LSE Health, London School of Economics and Political Science, London, United Kingdom

## Abstract

**Importance:**

Effective COVID-19 vaccines and therapeutics reached the market within the first year of the pandemic. This rate of development and availability was an unprecedented achievement that required attention to numerous research and development, regulatory, and policy challenges. However, only limited evidence is currently available on the sources of funding for COVID-19 clinical trials.

**Objective:**

To compare the number and funding sources of clinical trials aimed at investigating therapeutics and vaccines for COVID-19 vs those for all non–COVID-19 indications.

**Design, Setting, and Participants:**

In this cross-sectional study, clinical trials in phase 1 to 3 that were registered to start between January 1, 2020, and August 31, 2021, were examined. All relevant data were collected from ClinicalTrials.gov.

**Main Outcomes and Measures:**

Number of clinical trials and their funding sources.

**Results:**

A total of 1977 clinical trials that addressed COVID-19 therapeutics and vaccines were registered worldwide with starting dates from January 1, 2020, to August 31, 2021. This cohort represented 13.9% of all trials (N = 14 274) during the same period. Most of the COVID-19 therapeutic and vaccine clinical trials were funded by public sources (1144 [57.9%]), followed by industry (540 [27.3%]) and public-private partnerships (293 [14.8%]). Most of these studies focused on the development of anti–COVID-19 therapeutics (1680 [85.0%]) rather than vaccines (297 [15.0%]).

**Conclusions and Relevance:**

The findings of this study suggest that publicly funded research and medical institutions played a leading role as funding sources for generating effective COVID-19 therapeutics and vaccines during the first 1.5 years of the pandemic and were most likely instrumental in their rapid development. It may be beneficial for the public sector to maintain the affordability and global access to these therapeutics and vaccines to ensure that they remain available for use worldwide.

## Key Points

**Question:**

What were the major sources of funding for clinical trials focused on the development of therapeutics and vaccines against COVID-19 between January 1, 2020, and August 31, 2021?

**Findings:**

In this cross-sectional study of 1977 clinical trials, most were funded by public sources (57.9%), followed by industry (27.3%) and public-private partnerships (14.8%). Most of these clinical trials focused on COVID-19 therapeutics (85.0%) as opposed to vaccines (15.0%).

**Meaning:**

The findings of this study suggest that the public sector has likely been instrumental in the development of COVID-19 therapeutics and vaccines; efforts to maintain their global access and affordability would be beneficial for public health.

## Teaser

This cross-sectional study examines the funding sources of clinical trials for COVID-19 therapeutics and vaccines during the pandemic.

## Introduction

The COVID-19 pandemic resulted in profound challenges to global public health. Ongoing conditions have resulted in a massive deterioration of population health, the economy, and human well-being and have tested health systems’ preparedness and resilience. Numerous government-based public health interventions were introduced in an attempt to limit virus transmission in the community. Early measures included self-isolation, traffic restrictions, physical distancing, and centralized quarantines.^1^ Limits on gatherings, closure of educational institutions and nonessential businesses, the introduction of border restrictions, and efforts to increase the availability of personal protective equipment proved to be among the most effective nonpharmaceutical interventions introduced during the first year of the pandemic.^2,3^ The greater the strength of government interventions at this early stage, the more effective they proved to be.^4^ Apart from nonpharmaceutical interventions, early studies indicated an urgent need for safe and effective vaccines and therapeutics to combat the pandemic by preventing against and managing clinical cases of COVID-19.^5,6^ Research and development (R&D) of therapeutics and vaccines that were effective against COVID-19 became a major priority for global medical research and a topic of interest for the public at large.

Hundreds of COVID-19 vaccines and therapeutics are under clinical development at this time,^7^ with more than 2 dozen vaccines and nearly a dozen treatments for COVID-19 being approved or authorized for human use.^8,9^ New vaccines reached the market within the first year of the pandemic and numerous repurposed drugs were in the late stages of clinical development, representing an unprecedented historical achievement. Several policy challenges had to be addressed to accomplish these goals.^10^ Specific changes to R&D incentives were made possible by strong public-private partnerships (PPPs) that linked the efforts of government, industry, and academia with international efforts coordinated by nongovernmental organizations. The US National Institutes of Health–led Accelerating COVID-19 Therapeutic Interventions and Vaccines partnership^11^ and Operation Warp Speed that coordinated the efforts of the US Government and the private sector^12,13^ are leading examples of such PPPs. Similarly, the Coalition for Epidemic Preparedness Innovation is an example of an international nongovernmental organization funded by nonprofit organizations and numerous country governments.^14^

Recent studies focusing on COVID-19 R&D have called for more collaborative efforts to improve the evidence base in clinical research^15^ and optimize the efficient use of resources by investing in well-designed clinical trials with prospects of generating high-quality data.^16^ However, we have only a limited understanding of the funding sources for COVID-19 clinical trials.^17^ A quantitative assessment of the nature of funding sources may provide insight into the public contribution toward the development of new COVID-19 therapeutics and vaccines, with implications on coverage of and access to these interventions once they are introduced to the market. A clear assessment of the funding sources for COVID-19 clinical trials could provide critical insight for policy makers who need to address existing and future challenges relating to R&D incentives and patient access for a wider scope of therapeutic indications (eg, antibiotics).

In this study, we identified registered clinical trials focused on therapeutics and vaccines for COVID-19 together with their funding sources that were initiated between January 1, 2020, and August 31, 2021, and compared these findings with clinical trials for indications other than COVID-19 initiated during this same period. The study aimed to understand how the landscape of biopharmaceutical R&D was shaped by funding from public and industry sources as well as their partnerships during the first 1.5 years of the COVID-19 pandemic.

## Methods

### Design

The study used a cross-sectional design with a focus on clinical R&D activity required to bring new biopharmaceutical interventions to the market. Phase 4 trials (ie, postlicensing trials) were excluded from the study. The Strengthening the Reporting of Observational Studies in Epidemiology (STROBE) guideline for cross-sectional studies was used for reporting. This study was approved by the MSc Research Ethics Committee from the London School of Hygiene and Tropical Medicine.

### Data Source

Quantitative data from all clinical trials (other than phase 4) designed to investigate therapeutics and vaccines for COVID-19 and non–COVID-19 indications were retrieved from the ClinicalTrials.gov data repository. The sources of funding were collected from the *funded by* variable of the registry and were classified into industry, public (ie, nonindustry such as National Institutes of Health, US Federal Government), and their combination as a PPP. Trials specifying other in the *funded by* variable were evaluated further with a review of the *sponsors/collaborators* variable; we identified these studies as publicly sponsored (ie, primarily academic hospitals, universities, and research centers); thus, they were classified as public for this analysis.

Data were retrieved from the ClinicalTrials.gov database on September 1, 2021. Initially, we searched for all phase 1 to 3 trials, including those assigned a not applicable phase status, that listed a start date between January 1, 2020, and August 31, 2021. Because this study focused on interventional trials that investigated the outcomes of therapeutics and vaccines, trials that did not specify drug or biological in the *intervention* variable were excluded (eg, dietary interventions, procedures, devices, or diagnostic tests). Similarly, trials with a *primary purpose* specified as diagnostic, basic science, screening, or device were excluded. Identification of trials to be excluded based on their *intervention* or *primary purpose* was conducted independently by 2 of us (A.A. and C.S.A.). This process provided us with the overall sample of trials to be screened. To identify trials specifically focused on COVID-19, we collected entries that specified COVID and/or SARS-CoV-2 in the *conditions* and/or *title* variables (COVID-19 sample). The remaining entries were designated non–COVID-19 trials (non–COVID-19 sample); a subgroup of these trials that specified infectious diseases in the *report groups* variable was included as a non–COVID-19 infectious disease cohort (non-COVID-19 infectious disease sample). A flow diagram documenting the identification of clinical trials of interest from ClinicalTrials.gov is shown in eFigure 1 in the Supplement.

### Statistical Analysis

Analysis was conducted using unpaired 2-sided *t* tests to determine whether the differences in the number of trials focused on COVID-19 vs those for non–COVID-19 indications achieved statistical significance. Mann-Kendall tests were used to determine whether differences in any of the trends observed with respect to the number of trials over time reached statistical significance. Specifically, we determined whether the number of clinical trials both for COVID-19 and non–COVID-19 indications (including industry funded, nonindustry funded, and PPP funded) exhibited a monotonic downward or upward trend (ie, consistently decreasing or increasing over time). The null hypothesis of the Mann-Kendall test implies that there are no statistically significant monotonic trends over the specified period; a *P* value <.05 suggested a rejection of the null hypothesis for the time series under investigation. Stata, version 15 (StataCorp Inc) was used throughout the study.

## Results

Our search of ClinicalTrials.gov revealed that 1977 clinical trials for COVID-19 therapeutics and vaccines were registered worldwide with a starting date from January 1, 2020, to August 31, 2021, representing 13.9% of all trials (N = 14 274) over the same period. The numbers of clinical trials for COVID-19 (n = 1977), non–COVID-19 (n = 12 297), and non–COVID-19 infectious disease (n = 852) indications registered during each month of the study period are shown in the Figure.

**Figure.**
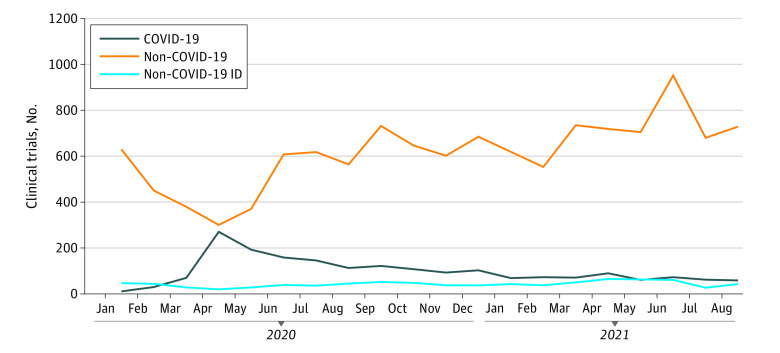
Number of Clinical Trials for COVID-19, Non–COVID-19, and Non–COVID-19 Infectious Disease Indications Registered During Each Month (January 1, 2020, Through August 31, 2021) ID indicates infectious disease.

Our analysis of these data revealed that COVID-19 trial registrations peaked in April 2020 (n = 271, representing 47.4% of all trials initiated during this month). The number of COVID-19 trial registrations reached a plateau from May to August 2021 (range, 59-73 [7.1%-8.3% of all trials]). Our analysis noted the fewest trial registrations in April 2020 for non–COVID-19 (301 [52.6% of all trials]) and non–COVID-19 infectious disease (20 [3.5% of all trials]) indications. Registrations peaked in June 2021 for non–COVID-19 trials (953 [92.9% of all trials]) and in April 2021 for non–COVID-19 infectious disease trials (65 [8.0% of all trials]). We observed a statistically significant decreasing trend in the number of COVID-19 clinical trials throughout this period (*P* < .05). By contrast, our analysis revealed a statistically significant increasing trend in the number of non–COVID-19 trials (*P* < .005) over this same period. No statistically significant trends were detected in our evaluation of non–COVID-19 infectious disease trials over time.

In terms of funding sources, of the 1977 clinical trials focused on COVID-19 indications, our analysis revealed that 1144 (57.9%) were publicly funded, 540 (27.3%) were industry funded, and 293 (14.8%) were funded via PPPs. Overall, 1680 (85.0%) of these studies were focused on the development of therapeutics and 297 (15.0%) on vaccines (Table 1). The public (ie, nonindustry) sector funded 1039 (61.8%) trials focused on therapeutic agents; only 413 (24.6%) of these trials were funded by the private sector (ie, industry). By contrast, vaccine trials were funded by 127 (42.8%) industry sources and 105 (35.4%) public sources. Public-private partnerships funded more vaccine trials than those focused on therapeutic agents (65 [21.9%] vs 228 [13.6%]). Overall, the public sector and PPPs together funded more than one-half of the vaccine trials (170 [57.3%]). The number of clinical trials and funding sources for COVID-19 therapeutics and vaccines registered each month vs those for non–COVID-19 indications are shown in eFigure 2 in the Supplement.

**Table 1.** Number of Clinical Trials for COVID-19 Therapeutics and Vaccines^a^

Trial	Industry funded	Publicly funded	PPP funded	Total (% of all COVID-19 trials)
All (% of all COVID-19 trials)	540 (27.3)	1144 (57.9)	293 (14.8)	1977 (100)
Therapeutics (% of COVID-19 trials focused on therapeutic agents)	413 (24.6)	1039 (61.8)	228 (13.6)	1680 (85.0)
Vaccines (% of COVID-19 trials focused on vaccines)	127 (42.8)	105 (35.4)	65 (21.9)	297 (15.0)

Abbreviation: PPP, public-private partnership.

^a^ Phase 1 to 3 trials, as well as clinical trials assigned a not applicable phase status, were included in the analysis.

The monthly breakdown of clinical trial registrations for COVID-19 according to funding source and type of intervention is presented in Table 2. With only 2 exceptions, we identified more publicly funded COVID-19 trials each month during this period than those funded by industry and PPPs (eFigure 2A in the Supplement). However, our analysis revealed a decreasing trend in both the number and relative share of publicly funded COVID-19 trials from 209 in April 2020 to 18 in August 2021, representing a decrease from 77.1% in April to 30.5% in August of all COVID-19 trials. Overall, our analysis of the results from January 1, 2020, through August 31, 2021, revealed a statistically significant decreasing trend in the number of publicly funded COVID-19 trials (*P <* .05). By contrast, our findings revealed a plateauing trend with respect to the number of industry-funded trials from April 2020 onward (20-42 trials). An evaluation of these findings, together with the decreasing number of publicly funded trials, revealed an increase in the relative contribution of industry-funded trials from 12.2% in April 2020 to 44.1% in August 2021 of all COVID-19 trials (eFigure 2A in the Supplement). However, no statistically significant trends were detected in the numbers of COVID-19 industry-funded or PPP-funded trials from January 2020 through August 2021.

**Table 2.** Number of Clinical Trials for COVID-19 Therapeutics and Vaccines^a^

Month	All clinical trials, No.	COVID-19 trials, No. (%)				COVID-19 therapeutic trials, No. (%)				COVID-19 vaccine trials, No. (%)			
		Total^b^	Industry funded^c^	Publicly funded^c^	PPP funded^c^	Total^c^	Industry funded^d^	Publicly funded^d^	PPP funded^d^	Total^c^	Industry funded^e^	Publicly funded^e^	PPP funded^e^
Jan 2020	642	11 (1.7)	0	10 (90.9)	1 (9.1)	11 (100)	0	10 (90.9)	1 (9.1)	0	0	0	0
Feb 2020	481	30 (6.2)	1 (3.3)	26 (86.7)	3 (10.0)	29 (96.7)	1 (3.4)	25 (86.2)	3 (10.3)	1 (3.3)	0	1 (100)	0
Mar 2020	450	70 (15.6)	8 (11.4)	58 (82.9)	4 (5.7)	65 (92.9)	8 (12.3)	54 (83.1)	3 (4.6)	5 (7.1)	0	4 (80.0)	1 (20.0)
Apr 2020	572	271 (47.4)	33 (12.2)	209 (77.1)	29 (10.7)	265 (97.8)	30 (11.3)	207 (78.1)	28 (10.6)	6 (2.2)	3 (50.0)	2 (33.3)	1 (16.7)
May 2020	564	193 (34.2)	30 (15.5)	134 (69.4)	29 (15.0)	183 (94.8)	27 (14.8)	129 (70.5)	27 (14.8)	10 (5.2)	3 (30.0)	5 (50.0)	2 (20.0)
Jun 2020	768	159 (20.7)	36 (22.6)	90 (56.6)	33 (20.8)	146 (91.8)	33 (22.6)	85 (58.2)	28 (19.2)	13 (8.2)	3 (23.1)	5 (38.5)	5 (38.5)
Jul 2020	765	146 (19.1)	42 (28.8)	91 (62.3)	13 (8.9)	129 (88.4)	37 (28.7)	82 (63.6)	10 (7.8)	17 (11.6)	5 (29.4)	9 (52.9)	3 (17.6)
Aug 2020	678	113 (16.7)	31 (27.4)	64 (56.6)	18 (15.9)	96 (85.0)	22 (22.9)	61 (63.5)	13 (13.5)	17 (15.0)	9 (52.9)	3 (17.6)	5 (29.4)
Sep 2020	855	122 (14.3)	39 (32.0)	62 (50.8)	21 (17.2)	101 (82.8)	30 (29.7)	54 (53.5)	17 (16.8)	21 (17.2)	9 (42.9)	8 (38.1)	4 (19.0)
Oct 2020	756	108 (14.3)	35 (32.4)	56 (51.9)	17 (15.7)	93 (86.1)	27 (29.0)	51 (54.8)	15 (16.1)	15 (13.9)	8 (53.3)	5 (33.3)	2 (13.3)
Nov 2020	696	93 (13.4)	33 (35.5)	44 (47.3)	16 (17.2)	80 (86.0)	29 (36.3)	39 (48.8)	12 (15.0)	13 (14.0)	4 (30.8)	5 (38.5)	4 (30.8)
Dec 2020	789	103 (13.1)	34 (33.0)	52 (50.5)	17 (16.5)	79 (76.7)	21 (26.6)	47 (59.5)	11 (13.9)	24 (23.3)	13 (54.2)	5 (20.8)	6 (25.0)
Jan 2021	689	69 (10.0)	23 (33.3)	32 (46.4)	14 (20.3)	58 (84.1)	17 (29.3)	29 (50.0)	12 (20.7)	11 (15.9)	6 (54.5)	3 (27.3)	2 (18.2)
Feb 2021	627	73 (11.6)	30 (41.1)	32 (43.8)	11 (15.1)	51 (69.9)	21 (41.2)	25 (49.0)	5 (9.8)	22 (30.1)	9 (40.9)	7 (31.8)	6 (27.3)
Mar 2021	807	71 (8.8)	29 (40.8)	29 (40.8)	13 (18.3)	45 (63.4)	18 (40.0)	19 (42.2)	8 (17.8)	26 (36.6)	11 (42.3)	10 (38.5)	5 (19.2)
Apr 2021	810	90 (11.1)	33 (36.7)	41 (45.6)	16 (17.8)	70 (77.8)	26 (37.1)	34 (48.6)	10 (14.3)	20 (22.2)	7 (35.0)	7 (35.0)	6 (30.0)
May 2021	767	61 (8.0)	26 (42.6)	27 (44.3)	8 (13.1)	42 (68.9)	16 (38.1)	20 (47.6)	6 (14.3)	19 (31.1)	10 (52.6)	7 (36.8)	2 (10.5)
Jun 2021	1026	73 (7.1)	31 (42.5)	32 (43.8)	10 (13.7)	56 (76.7)	23 (41.1)	25 (44.6)	8 (14.3)	17 (23.3)	8 (47.1)	7 (41.2)	2 (11.8)
Jul 2021	743	62 (8.3)	20 (32.3)	37 (59.7)	5 (8.1)	45 (72.6)	12 (26.7)	30 (66.7)	3 (6.7)	17 (27.4)	8 (47.1)	7 (41.2)	2 (11.8)
Aug 2021	789	59 (7.5)	26 (44.1)	18 (30.5)	15 (25.4)	36 (61.0)	15 (41.7)	13 (36.1)	8 (22.2)	23 (39.0)	11 (47.8)	5 (21.7)	7 (30.4)
Total	14 274	1977 (13.9)	540 (27.3)	1144 (57.9)	293 (14.8)	1680 (85.0)	413 (24.6)	1039 (61.8)	228 (13.6)	297 (15.0)	127 (42.8)	105 (35.4)	65 (21.9)

Abbreviation: PPP, public-private partnership.

^a^ Phase 1 to 3 trials, as well as clinical trials assigned a not applicable phase status, were included in the analysis.

^b^ Percent of all trials.

^c^ Percent of all COVID-19 trials.

^d^ Percent of all COVID-19 therapeutic trials.

^e^ Percent of all COVID-19 vaccine trials.

A similar analysis for non–COVID-19 indications (n = 12 297) is reported in Table 3. The percentage of all publicly funded trials for non–COVID-19 indications was lower (46.1%) and that of industry-funded trials was higher (40.7%) than the fraction of studies funded for COVID-19 applications. The fraction of non–COVID-19 trials funded by PPPs (13.2%) was also lower than those funded for COVID-19 indications. These differences were statistically significant (*P* < .001). Our findings revealed statistically significant increasing trends in the number of publicly funded (*P* < .01), industry-funded (*P* < .005), and PPP-funded (*P* < .005) non-COVID-19 clinical trials over time (January 2020-August 2021), with peak numbers observed at June 2021 and nadirs at April 2020 (eFigure 2B in the Supplement). The relative contributions of public and industry funding to the total number of non–COVID-19 trials were less divergent than were those to COVID-19 trials. For non–COVID-19 trials, the percentage of publicly funded trials ranged from 39.5% (December 2020) to 51.5% (January 2020), and the number of trials funded by industry ranged from 35.0% (January 2020) to 45.6% (July 2020) (eFigure 2B in the Supplement).

**Table 3.** Number of Clinical Trials for Non–COVID-19 Indications^a^

Month	All trials, No.	Non-COVID-19 trials, No. (%)			
		Total^b^	Industry funded^c^	Publicly funded^c^	PPP funded^c^
Jan 2020	642	631 (98.3)	221 (35.0)	325 (51.5)	85 (13.5)
Feb 2020	481	451 (93.8)	173 (38.4)	218 (48.3)	60 (13.3)
Mar 2020	450	380 (84.4)	139 (36.6)	195 (51.3)	46 (12.1)
Apr 2020	572	301 (52.6)	109 (36.2)	146 (48.5)	46 (15.3)
May 2020	564	371 (65.8)	135 (36.4)	183 (49.3)	53 (14.3)
Jun 2020	768	609 (79.3)	243 (39.9)	287 (47.1)	79 (13.0)
Jul 2020	765	619 (80.9)	282 (45.6)	263 (42.5)	74 (12.0)
Aug 2020	678	565 (83.3)	241 (42.7)	249 (44.1)	75 (13.3)
Sep 2020	855	733 (85.7)	325 (43.0)	343 (46.8)	75 (10.2)
Oct 2020	756	648 (85.7)	256 (39.5)	311 (48.0)	81 (12.5)
Nov 2020	696	603 (86.6)	265 (43.9)	248 (41.1)	90 (14.9)
Dec 2020	789	686 (86.9)	306 (44.6)	271 (39.5)	109 (15.9)
Jan 2021	689	620 (90.0)	221 (35.6)	310 (50.0)	89 (14.4)
Feb 2021	627	554 (88.4)	234 (42.2)	266 (48.0)	54 (9.7)
Mar 2021	807	736 (91.2)	311 (42.3)	327 (44.4)	98 (13.3)
Apr 2021	810	720 (88.9)	301 (41.8)	306 (42.5)	113 (15.7)
May 2021	767	706 (92.0)	285 (40.4)	319 (45.2)	102 (14.4)
Jun 2021	1026	953 (92.9)	364 (38.2)	462 (48.5)	127 (13.3)
Jul 2021	743	681 (91.7)	294 (43.2)	313 (46.0)	74 (10.9)
Aug 2021	789	730 (92.5)	310 (42.5)	322 (44.1)	98 (13.4)
Total	14 274	12 297 (86.1)	5005 (40.7)	5664 (46.1)	1628 (13.2)

Abbreviation: PPP, public-private partnership.

^a^ Phase 1 to 3 trials, as well as clinical trials assigned a not applicable phase status, were included in the analysis.

^b^ Percent of all trials.

^c^ Percent of all non–COVID-19 trials.

We reevaluated our findings based on a subgroup of non–COVID-19 trials that focused specifically on infectious disease indications (n = 852). Our analysis revealed that the percentage of publicly funded trials for non–COVID-19 infectious disease indications was lower (48.0%) and that of industry-funded trials was higher (37.6%) than that provided for COVID-19 trials. Similarly, the percentage of PPP-funded trials was slightly lower (14.4%) than that provided for COVID-19 indications (Table 4). These differences reached statistical significance (*P* < .001). No statistically significant trends were detected in the numbers of publicly funded, industry-funded, or PPP-funded non–COVID-19 infectious disease clinical trials during this period (all *P* > .05). However, we found that the distribution of the relative shares of publicly funded non–COVID-19 infectious disease trials was narrower over time than that observed for COVID-19 trials, ranging from 30.0% (April 2020) to 71.4% (March 2020). The percentages of industry-funded non–COVID-19 infectious disease trials varied nearly as much as did those for COVID-19 trials, ranging from 17.9% in March to 60.7% in May 2020 (eFigure 2C in the Supplement).

**Table 4.** Number of Clinical Trials for Non–COVID-19 Infectious Disease Indications^a^

Month	All trials, No.	Non-COVID-19 infectious disease trials, No. (%)			
		Total^b^	Industry funded^c^	Publicly funded^c^	PPP funded^c^
Jan 2020	642	47 (7.3)	14 (29.8)	27 (57.4)	6 (12.8)
Feb 2020	481	44 (9.1)	18 (40.9)	21 (47.7)	5 (11.4)
Mar 2020	450	28 (6.2)	5 (17.9)	20 (71.4)	3 (10.7)
Apr 2020	572	20 (3.5)	10 (50.0)	6 (30.0)	4 (20.0)
May 2020	564	28 (5.0)	17 (60.7)	9 (32.1)	2 (7.1)
Jun 2020	768	39 (5.1)	18 (46.2)	15 (38.5)	6 (15.4)
Jul 2020	765	36 (4.7)	19 (52.8)	13 (36.1)	4 (11.1)
Aug 2020	678	45 (6.6)	17 (37.8)	22 (48.9)	6 (13.3)
Sep 2020	855	52 (6.1)	22 (42.3)	19 (36.5)	11 (21.2)
Oct 2020	756	48 (6.3)	16 (33.3)	26 (54.2)	6 (12.5)
Nov 2020	696	38 (5.5)	17 (44.7)	17 (44.7)	4 (10.5)
Dec 2020	789	37 (4.7)	17 (45.9)	13 (35.1)	7 (18.9)
Jan 2021	689	43 (6.2)	16 (37.2)	23 (53.5)	4 (9.3)
Feb 2021	627	38 (6.1)	12 (31.6)	20 (52.6)	6 (15.8)
Mar 2021	807	50 (6.2)	20 (40.0)	26 (52.0)	4 (8.0)
Apr 2021	810	65 (8.0)	21 (32.3)	36 (55.4)	8 (12.3)
May 2021	767	63 (8.2)	19 (30.2)	30 (47.6)	14 (22.2)
Jun 2021	1026	61 (5.9)	17 (27.9)	32 (52.5)	12 (19.7)
Jul 2021	743	27 (3.6)	10 (37.0)	14 (51.9)	3 (11.1)
Aug 2021	789	43 (5.4)	15 (34.9)	20 (46.5)	8 (18.6)
Total	14 274	852 (6.0)	320 (37.6)	409 (48.0)	123 (14.4)

Abbreviation: PPP, public-private partnership.

^a^ Phase 1 to 3 trials, as well as clinical trials assigned a not applicable phase status, were included in the analysis.

^b^ Percent of all trials.

^c^ Percent of all non–COVID-19 infectious disease trials.

## Discussion

Our analysis of 1977 therapeutic and vaccine clinical trials for COVID-19 indications revealed that most of these studies were funded by public sources (57.9%) and focused on the development of therapeutics (85.0%); these findings are consistent with earlier evidence.^17^ However, funding sources for therapeutics and vaccine trials differed to some extent. Of the 1680 therapeutic trials evaluated in our study, 61.8% were publicly funded and only 24.6% were funded by industry. In contrast, of the 297 vaccine trials, 42.8% were industry funded and 35.4% were publicly funded. This difference may relate to more funds being available for vaccine development via PPPs and the fact that vaccines are likely to be more profitable and thus of greater interest to industry, as well as the public support provided for more than 400 therapeutic trials conducted on repurposed drugs.^18^

Our evaluation of both COVID-19 and non–COVID-19 clinical trials and their sources of funding during the study period revealed several important trends. First, our findings suggest that public funders provided the earliest investments in COVID-19 trials; this trend was followed by funding from industry sources. The difference between the relative share of publicly funded vs industry-funded COVID-19 clinical trials (57.9% vs 27.3%) was larger than their respective differences for non–COVID-19 (46.1% vs 40.7%) and non–COVID-19 infectious disease (48.0% vs 37.6%) indications. Also, the overall decreasing trend observed in the number of registered COVID-19 clinical trials over time can be attributed to the decreasing trend in the number of publicly funded trials, as no significant similar downward trends were observed in the numbers of industry-funded or PPP-funded clinical trials.

Taken together, our results indicate that the public sector was most likely instrumental in the development of COVID-19 vaccines and therapeutics. Thus, it would be critical to determine ways to safeguard both the affordability and global access to these health-sustaining modalities.^19,20^ The public sector facilitated critical R&D investments and provided funding that extended beyond basic research to include late-stage clinical development,^21^ notably during the early phases of the pandemic.

Our results suggest that the number of COVID-19 trials at any given time may influence the number of non–COVID-19 trials. For example, the peak of COVID-19 clinical trial registrations and the nadir of non–COVID-19 and non–COVID-19 infectious disease clinical trial registrations both occurred in April 2020. These peak and nadir numbers for COVID-19 and non–COVID-19 clinical trials appear to have contributed to their respective decreasing and increasing trends observed throughout the study period. This observation is consistent with recent findings^22^ documenting reduction in trial activations for non–COVID-19 indications during the initial pandemic period (February 2020-May 2020) as the number of COVID-19 trials increased. Specifically, Unger and Xiao^22^ reported a 43% reduction in the per-month initiation of US-based trials overall and a 23% reduction in the per-month initiation of non–US-based trials. Reduced rates of initiation of clinical trials for other diseases could ultimately have negative implications on the overall development of new therapeutics and vaccines and thus patient health outcomes. Results from a similar study^23^ that examined enrollment in clinical trials for cancer-related indications during the first year of the COVID-19 pandemic revealed a steep decrease during the initial COVID-19 wave and an overall 23% decrease in enrollment. More precisely, this study indicated a 46% reduction in enrollment for cancer control and prevention trials and a 9% reduction in enrollment for treatment trials. However, taken together, these results suggest that clinical research rapidly adapted to the ongoing pandemic and related circumstances.^23^

### Limitations

This study has limitations. The study did not capture R&D activities that preceded clinical trials (eg, basic research). Another limitation relates to the use of the ClinicalTrials.gov repository as the only source of data. Although it is the most comprehensive data repository of its kind, studies registered with ClinicalTrials.gov frequently do not include all of the information needed for a comprehensive study of this nature; some trials are missing from the database, and specific information may be missing from individual records. Furthermore, records can be modified at any time (ie, additions, editing, and even deletions).^24^

## Conclusions

Nearly 2000 clinical trials focusing on the development of COVID-19 therapeutics and vaccines were initiated during the first 1.5 years of the pandemic. Most of these trials were funded by public sources and investigated potential therapeutic agents. Publicly funded research and medical institutions played a leading role during the early stages of the pandemic, and their efforts appear to have been instrumental toward the rapid development of effective therapeutics and vaccines. However, the quality of clinical trials and the level of funding provided by public and private sources may differ substantially, which could have a large impact. Further work will be needed to understand the contributions of the public and private sectors toward the development of COVID-19 therapeutics and vaccines.

## Notes

Supplement. **eFigure 1.** Flow Diagram for the Identification of COVID-19 Therapeutics and Vaccines Clinical Trials **eFigure 2.** Monthly Numbers of Therapeutics and Vaccines Clinical Trials and Their Funding Sources for COVID-19, Non-COVID-19, and Non-COVID-19 Infectious Disease Indications
