# Supplementary material for: Funding Sources of Therapeutic and Vaccine Clinical Trials for COVID-19 vs Non–COVID-19 Indications, 2020-2021
Source: JAMA Netw Open. 2022 Aug 16;5(8):e2226892. doi: 10.1001/jamanetworkopen.2022.26892 (PMC9382437; doi:10.1001/jamanetworkopen.2022.26892)
Supplement: Supplement. — eFigure 1. Flow Diagram for the Identification of COVID-19 Therapeutics and Vaccines Clinical Trials eFigure 2. Monthly Numbers of Therapeutics and Vaccines Clinical Trials and Their Funding Sources for COVID-19, Non-COVID-19, and Non-COVID-19 Infectious Disease Indications [file jamanetwopen-e2226892-s001.pdf]

Supplemental Online Content

Angelis A, Suarez Alonso C, Kyriopoulos I, Mossialos E. Funding sources of therapeutic and vaccine clinical trials for COVID-19 vs non–COVID-19 indications, 2020-2021. *JAMA Netw Open.* 2022;5(8):e2226892. doi:10.1001/jamanetworkopen.2022.26892

eFigure 1. Flow Diagram for the Identification of COVID-19 Therapeutics and Vaccines Clinical Trials

eFigure 2. Monthly Numbers of Therapeutics and Vaccines Clinical Trials and Their Funding Sources for COVID-19, Non-COVID-19, and Non-COVID-19 Infectious Disease Indications

This supplemental material has been provided by the authors to give readers additional information about their work.

© 2022 Angelis A et al. *JAMA Network Open.*

eFigure 1: Flow diagram for the identification of COVID-19 therapeutics and vaccines clinical trials

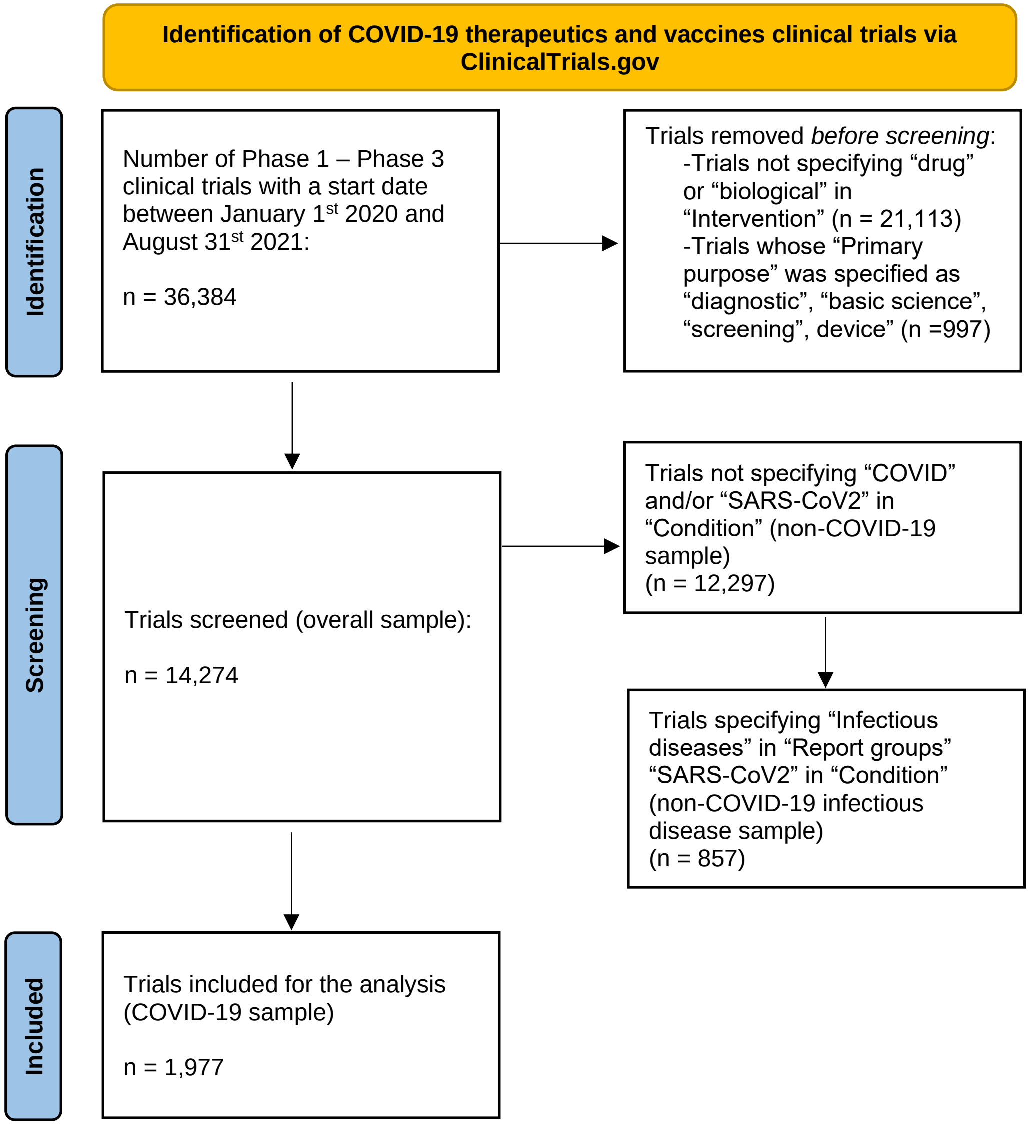

© 2022 Angelis A et al. *JAMA Network Open.*

eFigure 2: Monthly numbers of therapeutics and vaccines clinical trials and their funding sources for COVID-19 (a), non-COVID-19 (b) and non-COVID-19 infectious disease (c) indications

(a)

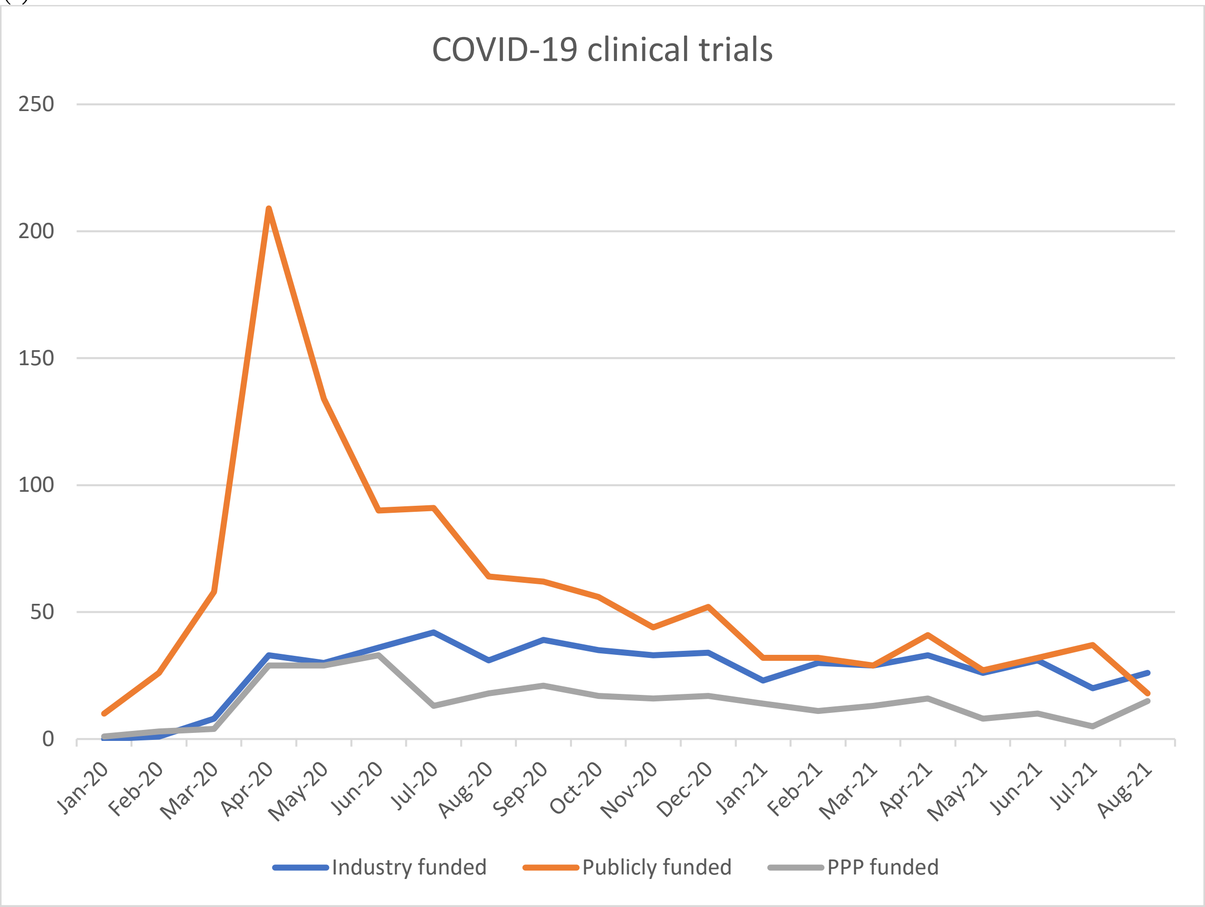

© 2022 Angelis A et al. *JAMA Network Open.*

(b)

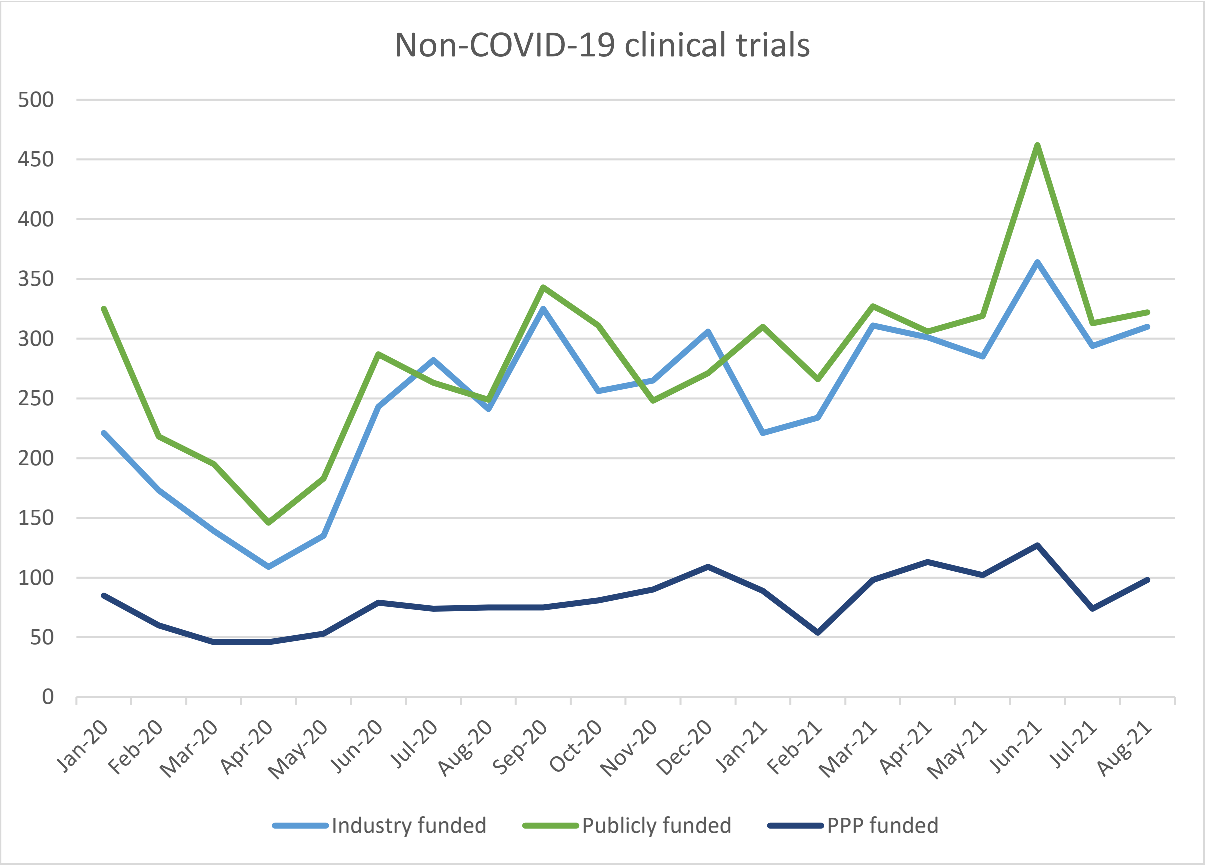

© 2022 Angelis A et al. *JAMA Network Open.*

(c)

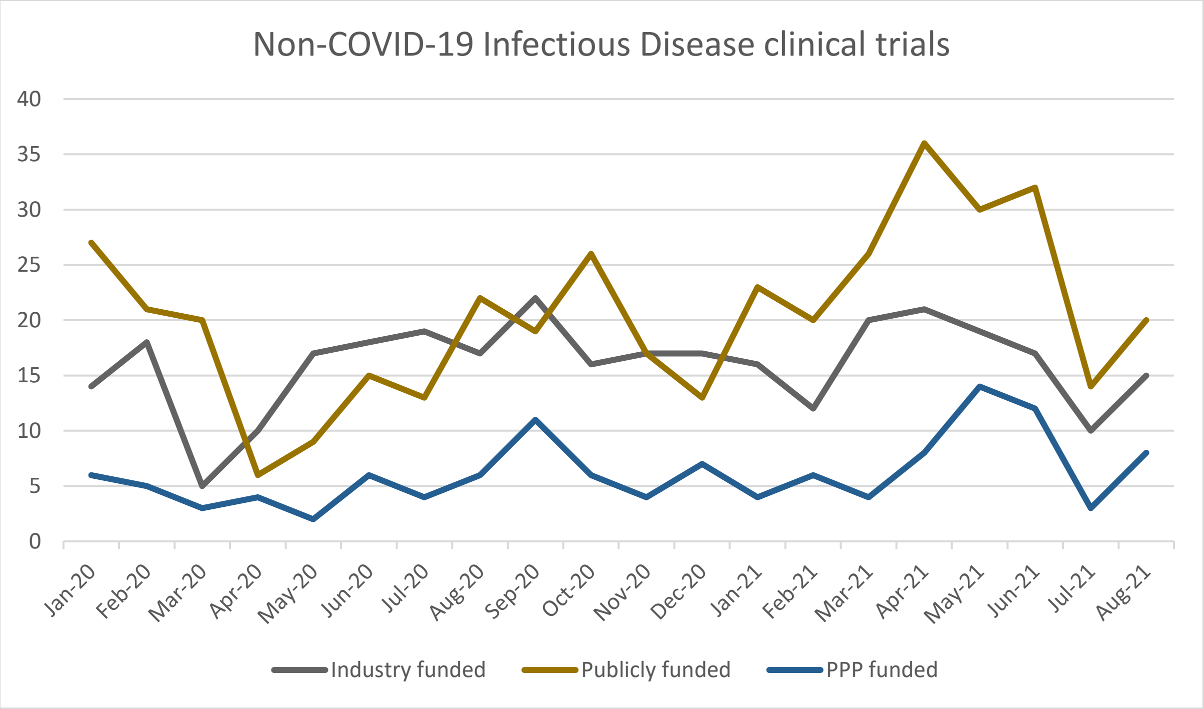

Note: Clinical trials assigned a "not applicable" phase status were also included.

© 2022 Angelis A et al. *JAMA Network Open*.
